# Study protocol for a pragmatic cluster RCT on the effect and cost-effectiveness of Everyday Life Rehabilitation versus treatment as usual for persons with severe psychiatric disability living in sheltered or supported housing facilities

**DOI:** 10.1186/s13063-022-06622-0

**Published:** 2022-08-15

**Authors:** Maria Lindström, Lars Lindholm, Per Liv

**Affiliations:** 1 Department of Community Medicine and Rehabilitation, Umeå University, 901 87 Umeå, Sweden; 2 Department of Epidemiology and Global Health, Umeå University, Umeå, Sweden; 3 Department of Public Health and Clinical Medicine, Umeå university, 901 87 Umeå, Sweden

**Keywords:** ELR intervention, Community-based mental health, Severe mental illness, Health equity, Collaboration, Occupational therapy, Participation, Recovery, Person-centred, Quality of life

## Abstract

**Background:**

People with severe psychiatric disabilities and impaired autonomy, living in sheltered or supported housing facilities, often lead sedentary, solitary lives indoors and have significantly poorer health than others in the population. Meaningful everyday activities are important for the recovery towards an enrichening, agentic, social, and hopeful everyday life. The Everyday Life Rehabilitation (ELR) model—a person-centred activity- and recovery-oriented intervention—has shown positive outcomes in feasibility studies, and thus a randomised controlled trial (RCT) is required to establish the effectiveness of ELR, along with calculations of cost-effectiveness.

**Methods:**

The ELR-RCT is a pragmatic, two-parallel-armed cluster RCT evaluating the effect and cost-effectiveness of using ELR from two measurement points over 6 months (pre-post intervention) and in three waves over 3 years. The primary outcome is recovering quality of life (ReQoL) at 6 months, and the secondary outcome is self-perceived recovery and daily functioning (RAS-DS) at 6 months. Additionally, Goal Attainment Scaling (GAS) will be used for the intervention group. Power analysis has been conducted for primary outcome measure. The first wave will include an internal pilot, to be evaluated after 6 months, used as basis for decisions on updating the required sample size and any other need for adaptations before continuing with the full-scale RCT in the second and third wave. All municipalities within a geographic area in northern Sweden, with a minimum of one sheltered or supported housing facility for people with severe psychiatric or neuropsychiatric disability, including access to occupational therapy, will be enrolled. Participants will be block-randomised to receive ELR plus treatment as usual (TAU) or TAU alone for a control period. The control group will thereafter receive delayed ELR. Occupational therapists and housing staff will receive an educational package, manuals, and tools, as well as reflections with colleagues during the intervention period. Housing managers will receive questions for monthly follow-up and coaching with staff.

**Discussion:**

This is a protocol for both an internal pilot and full trial of the first RCT study using the ELR intervention model in sheltered or supported housing facilities, evaluating the effects together with cost-effectiveness.

**Trial registration:**

ClinicalTrials.gov NCT05056415. Registered on 24 September 2021.

**Supplementary Information:**

The online version contains supplementary material available at 10.1186/s13063-022-06622-0.

## Administrative information

Note: the numbers in curly brackets in this protocol refer to SPIRIT checklist item numbers. The order of the items has been modified to group similar items (see http://www.equator-network.org/reporting-guidelines/spirit-2013-statement-defining-standard-protocol-items-for-clinical-trials/).

**Table:** 

**Title {1}**	Study protocol for a pragmatic cluster RCT on the effect and cost-effectiveness of Everyday Life Rehabilitation versus treatment as usual for persons with severe psychiatric disability living in sheltered or supported housing facilities
**Trial registration {2a and 2b}**	ID: NCT05056415 [ClinicalTrials.gov] [registered after start of inclusion; 24 September 2021]
**Protocol version {3}**	1.0
**Funding {4}**	This trial is funded by the Swedish Research Council for Health, Working Life and Welfare (FORTE 2021-01391).
**Author details {5a}**	Maria Lindström: MDr/PhD, Department of Community Medicine and Rehabilitation, Umeå University, 901 87 Umeå, Sweden. Lars Lindholm: Professor, Department of Epidemiology and Global Health, Umeå University, Sweden. Per Liv: PhD, Department of Public Health and Clinical Medicine, Umeå university, 901 87 Umeå, Sweden.
**Name and contact information for the trial sponsor {5b}**	The trial is initiated by Maria Lindström (Principal Investigator): maria.lindstrom01@umu.se Contact information for the funding agency Swedish Research Council for Health, Working Life and Welfare (in Swedish FORTE): https://forte.se/en/
**Role of sponsor {5c}**	This is an investigator-initiated trial. The funding agency approved the research proposal, based on a peer-review process evaluating the scientific quality, design, and relevance. Apart from the peer-review process, the funding agency has no role in the study design, data-collection, data-management, analyses, interpretation of data, writing the manuscript, or in the decision for publication.

## Introduction

In Sweden, people with long-term severe psychiatric disability (SPD) are entitled to live in sheltered or supported housing facilities, when the disability is causing significant difficulties in daily life and thus requires extensive support or service [1, 2]. Unfortunately, however, living in sheltered or supported housing facility often results in further reduced autonomy and stigmatisation due to the institutionalisation process [3]. It is mandatory to offer integrated basic healthcare, including rehabilitation, within the housing facilities [4], and this is a complex process involving a multitude of factors. Collaboration between healthcare and social service is even more complex due to separate legislations, secrecy rules, roles, and responsibilities [5]. Interventions that are useful under these conditions therefore need to be developed and studied. Furthermore, intervention research design must handle all of the complexities in order to develop evidence-based knowledge. To deal with this, a manualised but individually flexible model for integrated healthcare rehabilitation in collaboration between occupational therapists (OTs), housing staff (HS), and the resident in supported or sheltered housing facilities—namely, Everyday Life Rehabilitation (ELR)—has been developed by the principal investigator [6–8] and tested in feasibility studies [9–12] aiming at personal recovery through meaningful everyday activities and participation for persons with SPD. The MRC guidelines for complex interventions [13] have been thoroughly applied in the development process, including programme theory and the feasibility studies, and now evidence is needed for implementation, thus requiring randomised controlled trial (RCT) studies. Therefore, we want to expand the design and go further with a cluster RCT built on a slightly revised manual of the ELR intervention, adding clarified focus on leadership, the tools for collaboration, a web-based version of the education material, and a cost-effectiveness perspective.

### Background and rationale {6a}—programme theory for the development of the ELR intervention

#### Evidence base and theory behind the problem

‘Everyday occupation’, here synonymous with being occupied in ‘meaningful activity’, is fundamental for all people and refers to engagement in meaningful acts of doing, e.g. looking after oneself, taking care of a home, enjoying life, contributing to society, and interacting with others [14–16]. People with SPD living in sheltered or supported housing facilities often lead sedentary lives with an impoverished everyday life including few meaningful everyday occupations [17]. They are also frequently affected by overwhelming symptoms, disengagement, and difficulties handling everyday life situations [18]. SPD often includes or results in low autonomy [19], personal agency [10], and reduced motivation [20]. Additionally, SPD is considered to involve stigmatising circumstances and stands discrediting within social interactions [21], exposing status loss, stereotyping, and discrimination [22] along with social as well as economic marginalisation [23]. We found that some HS also tend to align with the stigmatising and degrading perspectives [11].

Viewing the problems from a societal level, people with SPD and impaired autonomy have significantly poorer health than others in the population, while at the same time they do not always have access to health care on an equal basis [22, 24]. The relation between sedentary lifestyle and problems of somatic ill health, reduced global functioning, and quality of life among persons with SPD is also well known and has been addressed [25]. However, methods to tackle somatic ill health do not fully reach out to persons with SPD because there is a problem with having the drive to change an unhealthy lifestyle, particularly for persons with negative symptoms related to schizophrenia [26]. Persons with schizophrenia live about 20 years shorter than the general population [27]. Sedentary lifestyle has appeared as an independent risk factor for morbidity and mortality [28], and a high amount of sedentary time significantly increases the risk of type 2 diabetes, all-cause mortality, and the incidence as well as mortality of both cardiovascular disease and cancer [29]. Thus, inequity in health in the society is being sustained. Sub-institutionalisation [3, 11], lack of guidance in everyday life activities to reduce sedentary time, differing and unequal healthcare/rehabilitation efforts, and challenges regarding collaboration, together with sparse interventions for this target group and context, add to the inequity for people with SPD in Sweden.

#### Contextual and legislative framework—a gap between policy goals and TAU

In Sweden, and in sheltered or supported housing facilities, health care including rehabilitation must be provided and offered. These efforts are regulated by health care legislation [4] while efforts made by HS are mainly regulated by social acts [1, 2]. Thus, there are two areas of responsibility where professionals, in order to meet the legal requirements, must collaborate in their work with the respective residents [5]. Despite this, in some municipalities, there is no rehabilitation at all, and in some municipalities there are very limited efforts for these target groups, thus reflecting unequal care and rehabilitation. The open model for priorities [24] intends to create increased systematics in order to ensure that health care regulated by legislation is regarded as a guaranteed resource and that relatively more resources are allocated to the use of appropriate and effective care for people with the greatest need for care, which includes medical treatment, nursing, rehabilitation, and habilitation [4]. Internationally, similar arguments are forwarded, for instance, the spending by the NHS in England, emphasising that extra resources should be used for services that benefit groups with poorer health. Equity is such a criterion based on fairness.

#### Intervention development

Given the inequity and marginalisation of the target group and the scarcity of collaborative, integrated re/habilitative methods when working within this complex context, the ELR package was designed and developed, based on best evidence and experiences from users, praxis, and stakeholders, to meet these challenges and to improve and transform the re-/habilitation efforts towards person-centred, motivational, and activity- and recovery-oriented resources. In order to thoroughly define the intervention, the TIDieR checklist for the intervention has been used [30].

#### Programme theory of ELR

**Fig. 1:**
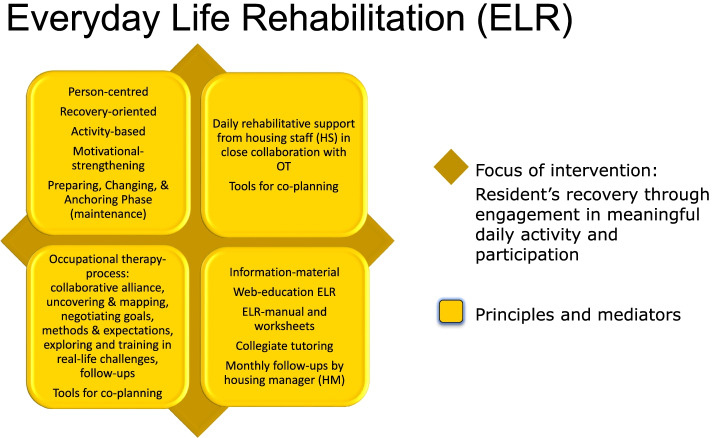
The Everyday Life Rehabilitation (ELR) model

ELR (Fig. 1) was constructed as an intervention model for integrated occupational therapy in sheltered and supported housing facilities [6–8], aiming at personal recovery through engagement in meaningful and enriching everyday activities for persons with SPD. The mediators identified from the best evidence and praxis, and combined in the ELR model, were person-centredness [16, 31, 32]; motivation strategies [33]; building a therapeutic alliance, empathy, and modulating the methods (tasks) to suit the specific person’s needs, expectations, and capacities [34, 35]; negotiation of user goal priority, planning, and expected outcome [36–38]; personal recovery [39–42]; engagement in meaningful activities [8, 14, 18, 43]; and methods for training in real-life activities and situations, led by OT; devices for close collaboration with residents and HS; support from HS on an everyday basis; and an educational package including tutorials, as well as collegial reflection and learning inspired by practice leadership [44].

The desired overall objectives of the intervention are based on robust paradigms for the target group’s health, wellbeing, and occupational justice ensured by personal recovery through enablement of engagement in meaningful everyday activities [14, 16, 18]. ELR is guided by an OT in close collaboration with participant, HS, and the social environment. Applying a preparation-, change-, and anchoring-phase (maintenance after goal attainment), the language and actions of professionals promote hope, self-discovery, meaning, connectedness, narrative reflection, transparent decisions shaped in partnership with residents, and exploring enriching everyday life activities.

#### Previous results from our feasibility studies of ELR prior to the RCT

A feasibility project with qualitative and quantitative studies was conducted [8–12] to evaluate perspectives of participants and professionals, indicating very promising tendencies, such as successful rehabilitation with goal-attainment, health, and re-engagement in home-based as well as social occupations, as described below.

One study [9] evaluated outcomes of the ELR intervention for residents (*n =* 17). Pre, post, and follow-up differences on goal attainment, occupation, and health-related factors indicated that important progress was made. We also carried out interviews and field observations (*n* = 16) after completing the ELR [10], thus disclosing participants’ stories of ‘rediscovering agency’, referring to occupational and identity transformations, and the mechanisms of the intervention, i.e. hope, extended value of reaching goals, re-entering general society, and the transparency of the process. Focus group interviews with 21 HS [11] illuminated their views on residents, rehabilitation, and their own role along with organisational conditions and different outlooks influencing their responsiveness or resistance to the intervention. Importantly, HS are a key resource in the facility context, but their roles and their views in facilitating or inhibiting rehabilitative opportunities for residents varied a lot. Narrative analysis of OTs’ stories [12] revealed ‘personalised occupational transformations’ describing complex processes and significant interactive events based on each resident’s wishes.

#### Theory of change processes

Low autonomous motivation has been identified in persons with SPD and negative symptoms [19, 26]. Because motivation is not only about inner will, autonomy, and agency, but also is greatly affected by the environment, that is, the people and conditions one is surrounded by, strategies in ELR are to a great extent about involving the social environment, that is, social network, HS and OTs, in supporting the person at a certain level of motivation and goal-ambitions with specific strategies and exploring enriching activities in order to gradually strengthen the inner will and desire for goal attainment. In order to obtain personalised occupational transformations [12], the OTs need to be individually flexible and tentative, and yet structured and transparent according to the goals and methods used. Because negotiated decision-making is an important method that facilitates motivation in person-centred care [45], and because individual goal setting is a useful tool to support motivation and overall rehabilitation [46] and because the recovery approach is based on personal preferences and sociality [40], these strategies add to one another positively. Overall, ELR is centred on enabling engagement in meaningful and enriching everyday activities to induce personal recovery through a collaborative, person-centred, motivational, and activity and recovery-oriented intervention.

To describe and support implementation of the present intervention package, a checklist for implementation has been used [47] to identify some crucial factors for this context based on the literature and on the feasibility studies. These include the difficult process of active acquisition of knowledge among leaders and personnel; attitudinal changes; capacity building; health care delivery and approach; the praxis of collaboration between OTs, HS, and participants; highly loaded HMs/distanced management; the importance of involving leaders in regular coaching and follow-ups of adherence; stigma; sub-institutionalisation; patient status; professional status; low motivation; and the differentiation among persons with SPD. In order to meet these challenges, the ELR package is constructed to be not too heavily loaded while still focusing on an enriching everyday life for the person. This is done via a shared model for staff where both the HM, housing, and rehabilitation staff, in the form of web-training, manuals, and guidance, can take part in a framework designed especially for these contexts. The ELR package gives them access to methods and tools for optimising the person’s opportunities to recovery through increased commitment to meaningful activities and participation in life, and for staff and management to learn through collegiate and reflective approach, inspired by practice leadership [44].

#### The ELR project as a whole

To summarise, the ELR-RCT is the next phase of research, based on the feasibility research conducted on ELR. The ELR-RCT will investigate the effects and costs of the intervention in order to generate evidence that may be transferable to similar settings. Besides the RCT, the ELR project as a whole will, over a 4-year period (2021–2025), rigorously evaluate the essential components, process factors, and impacts of ELR at multiple levels, including participants’ experiences, HS’s experiences, OTs’ experiences, HMs’ experiences, service outcomes, and implementation requirements. By studying outcomes as well as qualitative and process aspects, the ELR project asks not only *if* ELR works in these contexts, but also *how* it works in order to clarify practical and organisational guidance on the implementation of ELR in similar settings. These studies are not included in this RCT protocol. However, the continuing development of ELR manuals as well as its implementation will take into consideration aspects such as planning for organisational readiness, continued involvement of relevant stakeholders, and allowing for modifications.

#### Key uncertainties and justification for undertaking the trial

Initial evidence for the ELR model is based on positive outcomes in feasibility studies, and thus, an RCT is required to establish the effectiveness of ELR along with calculations of cost-effectiveness and continued process evaluations. Because of a lack of a formal control group, no effect size has been calculated. Therefore, this study will include an internal pilot to calculate the effect size after 6 months and to decide on relevant sample sizes and any need for adaptations before continuing with the full-scale RCT. In order to study how these health, contextual, and legal demands could be better fulfilled for people with SPD, we plan to apply a health economic perspective informed by an equity approach [48]. We align with the idea that putting the main focus on cost-effectiveness criteria, such as the demands laid out in the Act of Healthcare [4], will produce the most health gains from a given budget [49].

The specific research questions (RQs) are as follows:

- *RQ1*: What is the effectiveness of the ELR intervention on recovery, quality of life, everyday functioning, and goal attainment compared to TAU?
- *RQ2*: What is the incremental cost-effectiveness ratio (ICER) for ELR compared to TAU?

### Objectives and research questions {7}

The objectives of this RCT is therefore to investigate the effectiveness and cost-effectiveness of a person-centred and activity- and recovery-oriented intervention package for people with SPD living in sheltered or supported housing facilities.

### Trial design {8}

This study protocol covers the ELR-RCT, which is a pragmatic, two parallel arms, cluster RCT. The framework for present study is a superiority trial, and all statistical tests will be testing the null hypotheses that the two arms are equal.

The study has two measurement points over 6 months, including pre and post intervention (t1 = baseline, t2 = 6-month follow-up) in three waves over 3 years, where the first wave serves as an internal pilot study for the full trial. Randomisation will be performed separately at the three waves. The randomisation will be stratified on municipalities, giving a 1:1 allocating ratio of housing facilities within each participating municipality. As the number of participants within each housing facility will vary, the allocation ratio of participants in the study will not be fully 1:1 balanced. The design includes a waiting list as the control group, meaning that they will receive the ELR intervention after the control period. The protocol adheres to the SPIRIT statement, and the study will be conducted and reported in line with the Consolidated Standards of Reporting Trials (CONSORT) and the Consolidated Health Economic Evaluation Reporting Standards (CHEERS).

## Methods: participants, interventions, and outcomes

### Study setting {9}

The study will take place in the north of Sweden within geographic districts around Umeå. All municipalities agreeing to partake, with a minimum of one sheltered or supported housing facility for people with severe psychiatric or neuropsychiatric disability, including access to occupational therapy, will be enrolled. The coordinating centre is at Umeå University.

### Eligibility criteria {10}

Persons targeted in this study are adults with SPD living in sheltered or supported housing facilities [1, 2] in the participating municipalities. These persons may have a mixture of mental diagnoses, most commonly schizophrenia, other psychoses, personality disorders, affective disorders, severe neuropsychiatric disorders, and/or combined drug-related disorders.

#### Inclusion criteria

Inclusion criteria are adult residents (18 years of age or older) of the participating sheltered and supported housing facilities having a severe psychiatric or neuropsychiatric disability.

#### Exclusion criteria

All efforts will be made to include participants with communication and cognitive impairments because this will more accurately reflect the population characteristics. However, persons with dementia or severe developmental disability, not being able to communicate in Swedish, or currently being in acute psychosis or acute suicidal risk, will be excluded.

### Who will take informed consent? {26a}

Potential participants will, during a 2-month period prior to intervention, receive leaflets and oral information at their housing facilities, including initial study information. After reflection, they may either post their written informed consent to the trial administrator (UN) or attend to a meeting to discuss any remaining questions and sign the informed consent.

### Additional consent provisions for collection and use of participant data and biological specimens {26b}

It may be relevant with further follow-up later on, and participants have agreed to use data for such purposes.

### Interventions

#### Explanation for the choice of comparators {6b}

All participants will receive TAU. Participants allocated to the intervention group will additionally receive ELR during 6 months, which is a long-term, timed, personalised, and activity- and recovery-oriented rehabilitation process enabled by OTs and HS in collaboration via a shared framework and web education undertaken prior to the intervention.

#### Intervention description {11a}

##### TAU

The control is TAU, as provided at each of the respective municipalities. In the involved municipalities, TAU mainly consists of short-term efforts such as prescribing technical aids, often initiated by the HS to the OT. TAU also comprises a broad spectrum of non-medical daily residential services and psychosocial support provided by HS. Daily services and support by HS vary depending on the approach and commitment of individual staff and the norms that prevail in different housing units. Co-planning on long-term rehabilitation efforts does not exist or is weak, and collaboration between OTs and HS is, as described by staff from both parties, difficult to achieve.

##### The ELR intervention package

ELR has been developed as an intervention model for collaboration between OTs and HS in order to address the occupational imbalance and injustice of persons with SPD living in sheltered or supported housing facilities. Based on feasibility studies, the original ELR has been slightly revised and updated [50]. The ELR is a person-centred, motivational, and activity- and recovery-oriented intervention package with integrated occupational therapy in collaboration with HS and the participant built on principles, mediators, and certain process steps. ELR is manualised but allows for individualised content. The focus is on promoting personal agency and personal recovery while targeting engagement in meaningful and enriching everyday life activities through person-driven goals and exploration as well as training in real-life settings (Fig. 1).

The occupational therapy within ELR consists of a weekly session with an OT, followed by regular collaboration with HS who support the resident on a daily basis, which is in line with guidance given by the OT and input shared from the HS.

#### Strategies to improve adherence to intervention {11c}

The web training, which is the same for OTs and HS, consists of ten sections that can be viewed individually at any time when it suits one’s daily schedule. The ELR manual for HS contains the essentials of the concepts of person-centredness, motivation, and activity and recovery orientation together with strategies, methods, and tools to be used by the OTs and HS during the intervention and in collaboration in order to promote early involvement in enhancing strategies, collaboration, and daily support. The ELR manual for OTs additionally includes in-depth descriptions of certain occupational therapy methods, processes, and worksheets. Also, both the OTs and the HS working with the participants on a daily basis will together participate in collegial and reflective learning. Further, the HMs will lead monthly reflections with staff based on two given questions for each occasion focusing on the process along with good examples from staff demonstrating the quality of the efforts given to participants. Adherence to the ELR-protocol will be monitored by OTs.

#### Criteria for discontinuing or modifying allocated interventions {11b}

Participants can leave the trial at any time for any reason, without consequences. The participation can also be ended by the investigator or OT, if the participant is in acute psychosis or acute suicidal risk.

#### Relevant concomitant care permitted or prohibited during the trial {11d}

Concomitant psychopharmacological treatment is permitted during the trial. No specific concomitant interventions are prohibited during the trial but will be recorded.

#### Provisions for post-trial care {30}

Participants allocated to the control group (waiting list) will start receiving the ELR after completion of follow-up at 6 months.

### Outcomes {12}

#### Primary outcome

The primary outcome is self-perceived ‘recovering quality of life score’ at 6 months, assessed using the recovering quality of life (ReQoL) [51]. Quality-adjusted life years (QALYs) will be derived based on the ReQoL scores for cost-effectiveness calculations. The ReQoL-UI classification system comprises six mental health items and one physical health (PH) item. Conventional time-trade-off (TTO) was used to elicit utility values that are modelled to enable the generation of QALYs for use in cost-utility analysis of mental health interventions. A valuation survey with members of the UK public, representative in terms of age, gender, and region, was conducted using face-to-face interviewer administered TTO. Sixty-four health states were valued by 305 participants. Estimated utilities modelled for all health states ranged from −0.195 (state worse than dead) to 1 (best possible state) [52].

#### Secondary outcomes

The secondary outcome is self-perceived ‘recovery and daily functioning score’ at 6 months, assessed using the Recovery Assessment Scale—Domains & Stages (RAS-DS) [53]. Demographic data will also be collected. Additionally, goal-attainment score will be measured within only the intervention group using the Goal Attainment Scaling (GAS) [54].

### Sample size calculations {14}

The study is designed to detect a difference of 5 points on the ReQoL-scale. The minimum reliable change and minimum important difference has been suggested to be 10 points for ReQoL-20 [55]. Assuming a standard deviation of 10 [51], an average cluster size of 2 participants per housing facility and an intraclass correlation of 0.1, a total of 35 housing facilities in each group is required to reach a power of 80% when using a significance level of 5%. Varying the ICC from 0.05 up to 0.2 in the sample size calculation gives required facilities ranging from 33 to 38. From a starting point, the target sample size will be 35 housings in each house. However, the target sample size for the full-scale RCT is planned to be updated before the inclusion of housing facilities and participants in wave 2, based on outcome variability and intraclass correlation observed in the internal pilot. The sample size was estimated using the function *n4means* from the R package CRTSize [56].

### Participant timeline {13}

**Fig. 2:**
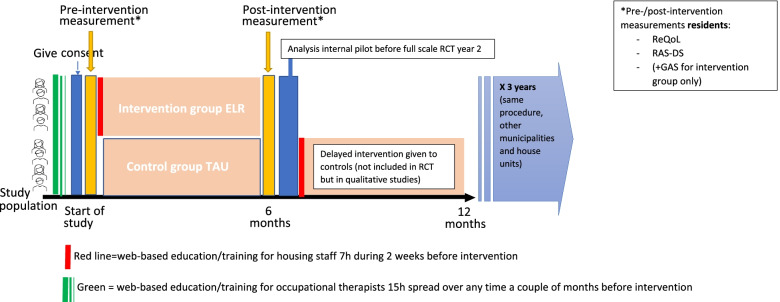
Timeplan and design of the RCT including internal pilot (first wave/year 1) and full scale RCT (second and third wave/years 2–3)

Figure 2 provides an overview of participant and trial’s timeline showing the major stages of enrolment, allocation, staff education, intervention period, and participant assessment points in line with the SPIRIT recommendations.

The recruitment period for the internal pilot began in the summer of 2021, data-collection t1 (baseline) took place between mid- and late August, web-education/training was ongoing between the 1st and 14th of September, and the intervention started on the 15th of September. This timeline will be repeated for years 2 and 3, that is the full RCT (Fig. 2).

### Recruitment {15}

Municipalities within the geographic area have been and will recurrently for 3 years be approached with information and an invitation to information meetings about the research project. Municipalities agreeing to participate, with a minimum of one sheltered or supported housing facility for people with severe psychiatric or neuropsychiatric disability, including access to occupational therapy, will be enrolled. Management, HMs, HS, OTs, and potential participants will be approached with written information about the study. Also, an oral explanation of the study and a leaflet describing the methods will be given prior to obtaining consent. The municipalities that agree to participate in the ELR study will be provided with a schedule and information material.

Recruitment of study participants will vary among clusters (housing units) due to differences in size, services, and participant criteria. Potential participants will be approached in different ways, such as leaflets, HS, OTs, HMs, and by word of mouth. All participants who give consent to participate and who meet the inclusion criteria will be included.

## Assignment of interventions: allocation

### Sequence generation {16a}, concealment mechanism {16b}, and implementation {16c}

**Fig. 3:**
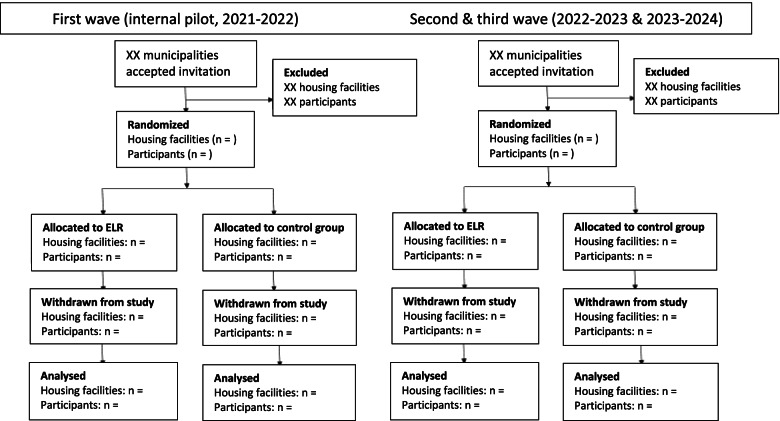
Draft of planned flow chart describing the enrolment, allocation, and analyses of participants and housing facilities in the publication of the study

Within the municipalities that have agreed to partake in ELR, sheltered and supported housing facilities meeting the inclusion criteria will be eligible for randomisation. Randomisation will be performed at housing-level, allocating all participants within the same housing facility to the same arm (Fig. 3). The allocation to either the intervention arm or control arm will be computer-generated by an external statistician (HH). The contact person of each municipality, the HMs, and the OTs will be informed in order to make the proper arrangements. The randomisation will be stratified on municipality, using a 1:1 allocation ratio to either ELR plus TAU or TAU alone for a control period of 6 months. The control group will thereafter receive delayed ELR plus TAU. The participants will be partly blinded, according to the information leaflet, indicating a ‘possible waiting list of six months’, that is, for the control group.

Professionals, that is, OTs and HS included in the intervention group, will receive passwords for web-based training during a 2-week period prior to the intervention and manuals about the ELR package, including tools to be used in intervention as well as tools for collaboration and collegial reflection over the intervention period. HMs will receive questions for monthly follow-up and coaching with staff. HS in the control group with TAU alone will not receive any education or manuals until the delayed ELR phase for the control group is offered, and their delayed intervention period will not be included in the full RCT calculations.

## Assignment of interventions: blinding

### Who will be blinded {17a}

Enrolment of participants will be completed by blinded assessors trained in Good Clinical Practice (GCP) and in working with people with SPD and/or the context of sheltered/supported housing facilities. They will assist in the completion of the consent process and will orally explain the study requirements, the privacy and ethical obligations of the research team, that participation is voluntary, and that information is kept private and locked. The blinded assessors will also support the participants in completing the demographic data sheet and the two self-assessment forms before and immediately after the 6-month period of either intervention or control—which will not be announced to either participants or blinded testers at the first measurement point, and will be kept secret from the blinded testers at the second measurement point. Thus, allocation will be concealed from the independent blinded assessors, and partly from the people with SPD participating in the intervention who will be informed that there may be a waiting list of 6 months for some participants. The treating OTs, HS, and HMs will, due to the character of the study, not be blinded to the intervention allocation.

Data will be blinded to the researchers until all analyses have been conducted. Trial statistician (PL) will be blinded for the analysis. A person at the university (UN) who is not involved in the interventions or the analysis will administrate and direct the blinded assessors and will collect and store the coded data in a locked fire-safety box.

Throughout the study, the randomisation will be conducted by an independent person (HH) at the Department of public health and clinical medicine, in order to keep the data management and statistician blind to the allocation for as long as the data bank is open.

### Procedure for unblinding if needed {17b}

As described above, allocation will be partly concealed from the participants/residents, who will be informed, prior to the study, that there may be a waiting list of 6 months for some participants. After the completion of baseline data-collection, they are informed of the date on which they will start ELR.

## Data collection and management

### Plans for assessment and collection of outcomes {18a}

The participant ReQoL-, RAS-DS-, and demographic data is collected under support and instructions, reminders, and explanations, of trained blinded testers. GAS data is collected under support and instructions of trained OTs. An independent person will monitor the GAS difficulty level in order to ensure that the participants do not choose too easy targets or change their goals after starting the intervention.

*ReQoL* [51] is a short and concise, valid and reliable, outcome measure focusing on the process of recovery and assesses the quality of life of people with different mental health conditions. ReQoL is comprised of positively and negatively worded items. Items cover areas of quality of life shown to be important for service users, including activity (meaningful); belonging and relationships; choice, control, and autonomy; hope; self-perception; well-being; and physical health. In ReQoL-20, the minimum score is 0 and the maximum is 80, where 0 indicates the poorest quality of life and 80 indicates the highest quality of life. Preference weights are available for the ReQoL to generate QALYs to be used in the cost-effectiveness evaluation of the intervention.

*RAS-DS* [53] is a valid and reliable measure of service user-defined recovery. A total recovery score is obtained from summing the scores for all 38 items. In RAS-DS, the minimum score is 0 and the maximum score is 152. It is also possible to have sub-score totals for each recovery domain. To calculate variance across domains, converting to percentages is recommended.

*GAS* [54] is a valid and reliable criterion-referenced measure that aids in quantifying the degree to which personal goals are attained. GAS enables the participant and OT to formalise and negotiate personalised goal setting, with a focus on challenging but realistic outcomes. Progress reflects meaningful change in a prioritised area. The GAS procedure includes documenting one or a set of participant-identified goals, identifying the construct of change that matters the most, and discussing what the outcome might look like if it is better or worse than the desired level of goal-attainment. If the participant has more than one goal, it is possible to weight each goal in terms of importance and difficulty, and thereafter use a formula that will calculate a T-score. The T-score or change in T-scores can be used to interpret the outcome of the intervention. If all of the goals meet the expected level of achievement, the GAS T-score will be 50. A high T-score (50 or above) is reflective of expected or higher goal attainment.

In addition, the OTs are responsible for reporting adherence to the intervention and process protocol and to the checklist for collaboration. The process protocols will be used also to collect data on resource use, for the cost-effectiveness calculations. The HMs are responsible for reporting monthly follow-ups with staff. The leads/contact persons of the municipalities are responsible for reporting any other departure from the protocol. Any deviations from the study protocol or the trial timeline, including withdrawals, or from the intervention manuals will be completely documented.

All data acquired during the trial will be anonymised and coded, saved in locked cabinets in locked rooms.

### Plans to promote participant retention and complete follow-up {18b}

Once participants are enrolled, practitioners as well as research workers will make every reasonable effort to follow them for the entire study period, including reminding them of the upcoming data collection and the benefits they will receive, especially once the control group is informed they are on the waiting list. Participants will receive continuous information about the study set-up and importance of follow-up. They are allowed to stop at any time without giving a reason. Trained, blinded testers will support data collection.

### Data management {19}

A data management plan (DMP) [57] is established, which describes how the research data are to be collected, stored, handled, documented, used, and made available during and after the research project. We will conduct this project in such a way as to protect the human rights and dignity of the participants, as reflected in the Helsinki Declaration. We will conform to GCP guidelines, data protection, and freedom of information acts. Storing of personal information and registration will follow the GDPR. All data will be stored securely in line with local data management arrangements. Personal identifiable paper records, such as informed consent, will be stored separate from anonymised paper records. All modifications made to the raw data and all steps taken in the analyses will be documented thoroughly.

### Confidentiality {27}

Only research staff, trial administrator (UN), and OTs of the municipalities will have access to the study data during the data-collection, analyses, and publication phase. Data on paper will be stored in lockable locations, and electronic data will be stored in password-protected locations, pseudonymised, or encrypted. Research data will be stored using a trial identification code for each participant. The code-list key will be documented and safeguarded by the trial administrator (UN) during data-collection, data analyses, and publication phase. Thereafter, the code-list key will be safeguarded by the PI, according to guidelines after the completion of the study.

### Plans for collection, laboratory evaluation, and storage of biological specimens for genetic or molecular analysis in this trial/future use {33}

Not applicable, no such collection will occur.

## Statistical methods

### Statistical methods for primary and secondary outcomes {20a}

Details about the statistical analyses of RCT outcomes can be found in the Statistical Analysis Plan (SAP); see Additional file 1. The SAP is also published at the trial’s site at clinicaltrials.gov (project-ID: NCT05056415).

#### Primary outcome measure (ReQoL)

A mixed-effects model will be used to estimate the effectiveness of ELR, and the dependent variable will be the ReQoL score at the 6-month follow-up. A random intercept effect for housing facilities will be included in the model. Fixed-effects independent variables will be *group* (ELR or control) and *baseline ReQoL measurement*. The baseline ReQoL will be adjusted for on the individual participant level and on the aggregated cluster level using the average of the baseline measurement within the corresponding housing facility. The treatment effect will be presented as the baseline-adjusted group effect at the 6-month follow-up along with its 95% confidence interval.

#### Secondary outcome measure (RAS-DS)

The same mixed-effects models will be used to analyse group differences in RAS-DS. The dependent variable will be the RAS-DS score at the 6-month follow-up. A random intercept effect for housing facilities will be included in the model. Fixed effects will be *group* (ELR or control) and *baseline RAS-DS measurement*. The baseline RAS-DS will be adjusted for on the individual participant level and on the aggregated cluster level using the average of the baseline measurement within the corresponding housing facility. The treatment effect will be presented as the baseline-adjusted group effect at 6 months’ follow-up along with its 95% confidence interval.

#### Secondary outcome measure (GAS)

GAS will be evaluated using paired *t*-tests to compare the pre- and post-intervention T-score.

### Interim analysis {21b}

After the first wave, the internal pilot will be analysed with respect to outcome variability, intra-class correlation drop-out rate, adherence, and required sample size for the full-scale study. While being an internal pilot, and hence its data will be included in the final analysis of full-scale study, the analysis at this stage will be non-comparative with respect to the study arms. Therefore, no adjustments of significance level in final analysis will be made. Should there be unexpected problems revealed by the internal pilot, e.g. feasibility problems or that it is shown that an unrealistic large sample size will be required for the study to be conclusive, there is opportunity to stop the study. This decision will be made by the investigators, and if so, the result from the internal pilot along with the motivation for stopping the study will be published. No official stopping rule has been predefined.

### Methods for additional analyses (e.g. subgroup analyses) {20b}

#### Sensitivity analyses

Analyses without imputation will be performed and reported in an Additional file 1 of the published study article.

#### Subgroup analyses

All analyses will be performed on men and women separately. Furthermore, sub-group analyses will be performed on:

- Autism/not autism
- Alcohol or drug addiction/no alcohol or drug addiction
- Psychosis-related/non-psychosis-related, based on self-reported diagnosis/disability

#### Analyses of cost-effectiveness

The trial is designed to measure effectiveness and cost-effectiveness. The latter is defined as the cost per QALY gained. The resources needed for the interventions will be measured in physical units (mainly time) and transformed into monetary values. Time used will be measured by interviews and diaries.

For cost-effectiveness analysis, the costs and effects of ELR plus TAU will be compared with TAU alone and will be presented as the ratio of incremental cost to incremental effect. Effects include health consequences as measured in QALYs. For this matter, ReQoL will be transformed into QALYs. Keetharuth and colleagues have developed an algorithm to transfer ReQoL into a preference-based scale that can be used for economic evaluations [58].

The Swedish Board of Health and Welfare has developed a model for priorities in health care that consists of the following criteria: severity of the condition, effectiveness of treatment, cost-effectiveness of treatment, and evidence base. The model assumes a specific condition linked to a specific treatment. This study is designed to provide the model with the proper data. A high degree of severity in these conditions has repeatedly been reported, and this will be investigated in this study. ReQoL and QALY weights are proper measures of severity.

When all sub-studies have been finalised, we plan to suggest a priority rank for the interventions being studied. The Board for Health and Welfare uses a scale in 10 steps, with 1 being the highest possible rank and 10 the lowest. This ranking is based on the four criteria described above.

### Methods in analysis to handle protocol non-adherence and any statistical methods to handle missing data {20c}

The primary analysis will use an intention-to-treat (ITT) approach and will include all allocated participants with valid data, whether they did or did not receive the complete intervention. For the primary ITT, missing data will be imputed using multiple imputation chained equations (MICE).

A per-protocol population will be defined as residents participating in at least 70% of the weekly sessions with OTs. Further criteria for adherence are HS completing the ELR education and HMs asking the monthly follow-up questions at staff meetings. The adherence will be summarised and presented groupwise in the publication of the study results.

The per-protocol population will be used for complementary and secondary analyses that will be presented in the study article.

### Plans to give access to the full protocol, participant-level data, and statistical code {31c}

The datasets will only be available to the research team and associated co-authors, during the trial analyses and publication phase. The datasets used can be made available by the PI, for review upon reasonable request, and in agreement with data transfer guidelines. Regarding reuse of data, it will be very limited, and decided by PI, due to the character of target group and sites.

## Oversight and monitoring

### Composition of the coordinating centre and trial steering committee {5d}

The trial is coordinated at the Umeå University. The independent administrator (UN) is responsible of the data collection, blinded testers, and safeguarding and storage of data, during data collection and analyses phases. Another person (HH), who is not involved in the study, independently manages the randomisation procedures.

The Trial Management & Steering Group (TMSG) includes the PI (ML), a statistician (PL), and a health-economist (LL), who are responsible for the trial design and adherence to the study protocol, trial registration, SAP, and the DMP. The TMSG meets approximately every month. The TMSG will have ultimate responsibility for the trial and can prematurely terminate the trial should unexpected problems be revealed by the internal pilot. If so, the results from the internal pilot along with the motivation for stopping the study will be published in a per-reviewed scientific article. No official stopping rule has been predefined.

The PI will ensure that the trial is conducted in line with GCP; takes supervision of the trial, trial registration, timeline, and overall quality; and coordinates the municipality anchoring. The PI is also in charge of communication with the leads/contact-persons of the municipalities, who in turn are responsible for the internal communication and scheme adherence within the municipal housing facilities; adherence to the timeline; the distribution of information provided to HMs, HS, and OTs; and the completion of tasks according to the trial scheme.

### Composition of the data monitoring committee, its role and reporting structure {21a}

We have no formal Data Safety and Monitoring Board, but the Umeå University organisation itself constitutes an oversight body independent of the sponsor and is responsible for data safety and quality issues, as well as for protecting the interests of the study participants.

### Adverse event reporting and harms {22}

All spontaneously reported adverse events will be recorded, assessed, and handled by the TMSG, in relation to potential harms, and unintended effects of trial interventions or trial conduct. Given prior experiences with the intervention, there is no reason to expect harms due to the study, but if any complications unexpectedly occur, we will report all adverse events in trial publications.

### Frequency and plans for auditing trial conduct {23}

No specific, independent party is designated for auditing the trial conduct. However, auditing can take place by regional health authorities. The proposed trial poses small risk to the participants. The interventions offered via ELR have shown preliminary evidence and do not expose the research subject to further risk or injury compared to TAU. Yet, the municipality delivery of and adherence to intervention is crucial. Also, the trial procedures are decisive. Therefore, the internal pilot during first wave will serve as a review, used as basis for decisions on updating the required sample size, investigating and improving feasibility, and any other need for adaptations before continuing with the full-scale RCT in the second wave.

### Plans for communicating important protocol amendments to relevant parties {25}

Any modifications of the protocol (e.g. after the internal pilot) that might impact on the conduct of the study, potential benefit of the residents, sample size calculations, or study procedures will be published as amendments to the present protocol article. Important protocol amendments will be communicated to trial participants, municipalities, and ethical committee. Minor changes in the protocol will be published at the trials site at clinicaltrials.gov. Violations of the study protocol will be included as an Additional file 1 to the published article of the final study.

### Dissemination plans {31a}

Trial outcomes will be published in open access, peer-reviewed scientific journals. Dissemination will also include conference papers and oral presentations, study updates for municipalities and the research community via the website of ELR [59], and knowledge transfer to government and the wider community through policy briefs and media where appropriate.

## Discussion

This study will be the first RCT using the ELR intervention model in sheltered or supported housing facilities, evaluating the effects together with cost-effectiveness. The ELR-RCT will provide the evidence required to eventually scale up and implement the intervention in more municipalities and similar settings and will indicate directions for future research on ELR.

Managing fruitful collaboration between OTs and HS, sharing the focus on supporting residents’ everyday life and daily activities, yet with different roles and responsibilities, is considered a main contributor to successful activity- and recovery-oriented rehabilitation in this context, but such collaboration is known to be challenging. Given the complex context, challenging collaboration between health and social care, the challenging situation of sedentary lifestyle and health inequities among residents, and the scarcity of both education and methods for managers and staff (HS and OTs) working in sheltered and supported housing facilities, the ELR package has potential to inform, inspire, guide, improve, and transform the re-/habilitation efforts towards person-centred, motivational, and activity- and recovery-oriented resources.

Recovery towards a meaningful and enrichening everyday life is crucial within sheltered and supported housing for persons with SPD. The key in ELR is person-centred, co-planned, and activity- and recovery-oriented mediation by listening, validating, encouraging, and enabling personal recovery through engagement in meaningful activity and participation.

ReQoL is a new outcome measure, with adequate psychometric properties [51], that applies to a continuum of all mental health disorders. We initiated the translation of ReQoL into Swedish for this project according to a rigorous procedure determined by the developer at the University of Sheffield, and we believe that ReQoL will be useful in several clinical and research frameworks on personal recovery. We chose ReQoL as the primary outcome measure because participant-reported recovery and quality of life are the key focuses of ELR and because it also enables the calculation of cost-effectiveness.

### Limitations

A cluster RCT can prove challenging in itself, and we believe even more so in the present context, particularly in low-resource settings with varying organisational structures. We expect, due to both previous feasibility studies [11] and the diverse type of leadership, norms, and attitudes among HS, that in some accommodations HS may show resistance to a new intervention model and thus also towards the web-education, manuals, worksheets, and the collaborating approach with the participants (residents) and OTs and thus deviate from the intended recovery-oriented path. Nevertheless, we argue for the importance of conducting an ELR-RCT in these particular types of housing facilities. Further, the natural setting entails a high likelihood of unexpected drop-out among accommodations, staff, and residents. There is also a probability for higher withdrawal among participants randomised to the control group once they are informed that they are on the waiting list. We still believe that a waiting list is best choice of method due to both the partial blinding during the control period and the motivation to agree to participate when being offered a ‘personalised rehabilitation period of six months with a possible waiting list of six month to start’.

Using GAS only for the intervention group is also a limitation, but this will still provide interesting data regarding within-group changes as in indicator of the response to treatment and the goal attainment of each participant.

### Trial status

This protocol is version 1, October 2021. Recruiting for the internal pilot, first wave, started in summer 2021, baseline data collection took place between 19th and 31st of August, training of staff in the experimental arm between 1st and 14th of September, and the intervention-period for participants started the 15th of September and will last for 6 months, ending with post-intervention-measurement. The approximate date when participant recruitment will be completed for the second and third wave, that is the full scale RCT, is 31st of August 2023. The third wave will continue until March 2024.

## Supplementary Information

**Additional file 1.** Statistical Analysis Plan.

## Data Availability

The trial datasets will be stored for 10 years. A data management plan (DMP) has been published. Principal Investigator/corresponding author (ML) is in charge of decisions regarding who will have access to the final trial dataset. Regarding authorship eligibility guidelines, the three co-authors of this study protocol will also be applicable in the trial publication. It may also be relevant with additional co-authors, which PI and the co-author group decide on. We do not intend to use professional writers.

## Abbreviations

DMP: Data management plan
DMSG: Data Management and Steering Group
ELR: Everyday Life Rehabilitation
GAS: Goal Attainment Scaling
GCP: Good Clinical Practice
HM: Housing manager
HS: Housing staff
ICER: Incremental cost-effectiveness ratio
ITT: Intention to treat
MICE: Multiple imputation chained equations
MRC: Medical Research Council
OT: Occupational therapist
PI: Principal Investigator
QALYs: Quality-adjusted life years
RCT: Randomised controlled trial
RAS-DS: Recovery Assessment Scale—Domains & Stages
ReQoL: Recovering quality of life
R&D: Research and Development units
RQ: Research question
SAP: Statistical Analysis Plan
SPD: Severe Psychiatric Disability
TAU: Treatment as usual
SFS: Swedish Code of Statuses
GDPR: General Data Protection Regulation
HSL: The Act of Healthcare
LSS: The Act concerning Support and Service for Persons with Certain Functional Impairments
PL: The Patient Act
PSL: The Patient Safety Act
SoL: The Social Services Act
