# Supplementary material for: Study protocol for a pragmatic cluster RCT on the effect and cost-effectiveness of Everyday Life Rehabilitation versus treatment as usual for persons with severe psychiatric disability living in sheltered or supported housing facilities
Source: Trials. 2022 Aug 15;23:657. doi: 10.1186/s13063-022-06622-0 (PMC9377097; doi:10.1186/s13063-022-06622-0)
Supplement: Supplementary file 1 — Additional file 1. Statistical Analysis Plan. [file 13063_2022_6622_MOESM1_ESM.pdf]

Statistical Analysis Plan

Effect and Cost-effectiveness of the Everyday Life Rehabilitation Intervention

A pragmatic RCT of Everyday Life Rehabilitation - a person-centered, recovery- and activity-based intervention package for residents with severe psychiatric disability living in sheltered or supported housing facilities

ClinicalTrials.gov identifier	NCT05056415
SAP version/date	1.0, 21-10-29
Principal investigator, co-author of SAP	Maria Lindström, PhD, Department of community medicine and Rehabilitation, Umeå university, Sweden
Responsible statistician, main author of SAP	Per Liv, PhD, Department of public health and clinical medicine, Umeå university, Sweden

SAP revisions

No revision has been made so far.

Signatures

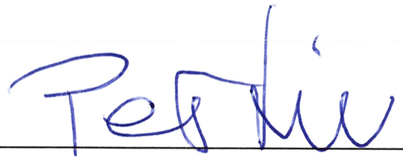

Per Liv, responsible statistician, and main author of SAP

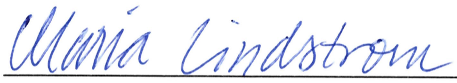

Maria Lindström, principal investigator and co-author of SAP

Content

SAP revisions 2
Signatures 2
Introduction 4
  Background and rationale 4
  Objectives 4
Study methods 4
  Trial design 4
  Randomization 4
  Sample Size 4
  Framework 5
  Statistical interim analysis and stopping guidance 5
  Timing of final analysis 5
  Timing of outcome assessments 5
Statistical principles 5
  Confidence intervals and *P* values 5
  Adherence and Protocol deviations 5
  Analysis populations 6
Trial Population 6
  Eligibility 6
  Recruitment 6
  Withdrawal/ Follow-up 6
  Baseline patient characteristics 7
Analysis 7
  Outcome definitions 7
  Analysis methods 8
  Missing data 8
  Additional analyses 9
  Statistical software 9
References 9

Introduction

The present statistical analysis plan (SAP) gives a detailed description of planned statistical analyses for the pragmatic cluster randomized controlled trial *Effect and Cost-effectiveness of the Everyday Life Rehabilitation*. The SAP is written with in consistency with the guidelines published by Gamle et al (1).

Background and rationale

People with severe psychiatric disability (SPD) frequently experience overwhelming symptoms, disengagement, and difficulties handling daily life situations. Generally, SPD includes or results in low autonomy and a sedentary lifestyle. The person-centered, motivational, recovery-, and activity-based intervention model `Everyday Life Rehabilitation´ (ELR), integrated in sheltered and supported housing facilities for people with SPD, has shown promising outcomes in feasibility studies (2–6) on health- and activity-measures for residents/participants, based on pre-post-follow-up measures of Satisfaction with Daily Occupations (SDO), Assessment of Motor- and Process-skills (AMPS), Social Interaction (BSI-II) (Assessment of Social Interaction Skills – II), ADL-taxonomy with a 5-grade scale on effort, Symptom Checklist – 90 items (SCL-90), Goal Attainment Scaling (GAS), as well as self-narrated changes in hope, identity, and personal agency. However, the effectiveness of ELR has not yet been established in a RCT.

Objectives

The overall objective of the present project is to investigate the effectiveness on quality of life, self-perceived recovery, everyday functioning, and goal-attainment of ELR for people with SPD living in sheltered or supported housing facilities.

Study methods

Trial design

The trial is pragmatic cluster randomized trial with two parallel arms. Housing facilities, serving as clusters of eligible participants, will be randomized into ELR or treatment as usual (TAU). Recruitment of participants and randomization will be performed in two waves, starting the intervention in September of 2021 and 2022, respectively. The study has an adaptive design, where data from the first wave of the study will be used in an internal pilot with the purpose of investigating and improving feasibility and updating sample size requirements before continuing with the full-scale RCT. The internal pilot data will be included in the analysis of the full-scale RCT.

Randomization

Randomization will be performed separately at the two waves. The randomization will be stratified on municipalities, giving a 1:1 allocating ratio of housing facilities within each participating municipality. This is partly due practical reasons; the required education of occupational therapists and housing staff could be unfeasible if too many housing facilities within a municipality are in the intervention group. As the number of participants within each housing facility will vary, the allocation ratio of participants in the study will not be fully 1:1 balanced.

The randomization is performed by an independent statistician, otherwise not involved in the project, by generating a group allocation for all included housing facilities using a computer script. The list of the housing facilities and participants will be completely anonymized for the statistician.

Sample Size

The study is designed to detect a difference of 5 points on the ReQoL-scale. The minimum reliable change and minimum important difference has been suggested to be 10 points for ReQoL-20.

Assuming a standard deviation of 10 (2), an average cluster size of 2 participants per hosting facility and an intraclass correlation of 0.1, a total of 35 housing facilities in each group is required to reach a power of 80% when using a significance level of 5%.

The target sample size is planned to be updated before the inclusion of housing facilities and participants in wave 2, based on outcome variability and intraclass correlation observed in the internal pilot.

Framework

The present study is a superiority trial, and all statistical tests will be of null hypotheses of the two arms being equal with respect to corresponding estimand.

Statistical interim analysis and stopping guidance

After the first wave, the internal pilot will be an analyzed with respect to outcome variability, intra-class correlation drop out rate, adherence and required sample size for the full-scale study. While being an internal pilot, and hence its data will be included in the final analysis of full-scale study, the analysis at this stage will be non-comparative with respect to the study arms. Therefore, no adjustments of significance level in final analysis will be made.
Should there be unexpected problems revealed by the internal pilot, e.g. feasibility problems or that it is shown that an unrealistic large sample size will be required for the study to be conclusive, there is opportunity to stop the study. This decision will be made by the investigators, and if so, the result from the internal pilot along with the motivation for stopping the study will be published. No official stopping rule has been predefined.

Timing of final analysis

All outcomes, primary and secondary, will be analyzed collectively after the locking of the database, when data collection is finished. The study project also involves cost effectiveness analyses of ELR. These analyses may be performed and published at different occasion than the analyses described in the present SAP.

Timing of outcome assessments

Measurements of ReQoL (primary outcome) RAS-DS (secondary outcome) will be conducted at baseline and at the 6-months follow-up, post-intervention. GAS, a secondary outcome, is only measurable for the intervention group at the 6-months follow-up.

Statistical principles

Confidence intervals and *P* values

A significance level of 5% will be employed in all hypothesis testing. The study includes one single primary outcome (ReQoL) and no adjustment to control for familywise type 1 error rate due to the multiple secondary outcomes will be performed. Precision of estimated parameters describing group differences will be assessed using 95% confidence intervals.

Adherence and Protocol deviations

Adherence to the intervention is defined as residents participating in at least 70% of the weekly sessions with occupational therapists. Further criteria for adherence are housing staffs completing

the ELR education and housing managers asking the monthly follow-up questions at staff meetings. The adherence will be summarized and presented groupwise in the publication of study results. Violations of the study protocol will be included as an appendix to the published article of the final study.

Analysis populations

The primary analysis will use an intention-to-treat (ITT) approach and include all allocated participants with valid data, whether they did or did not receive the complete intervention.

A per-protocol population will be defined as participants following the adherence criteria listed under Adherence and Protocol deviations paragraph will also be made. The per-protocol population will be used for complementary, secondary analyses and presented in the study article.

Trial Population

Eligibility

Eligible participants are adults with severe psychiatric disability (SPD), living in sheltered or supported housing facility for people with SPD with access to occupational therapy.

Exclusion criteria are comorbidity of dementia or severe developmental disability, not being able to communicate in Swedish and currently being in acute psychosis, or acute suicidal risk.

Recruitment

In Figure 2, the planned information that will be reported in the flow chart of study participants is shown.

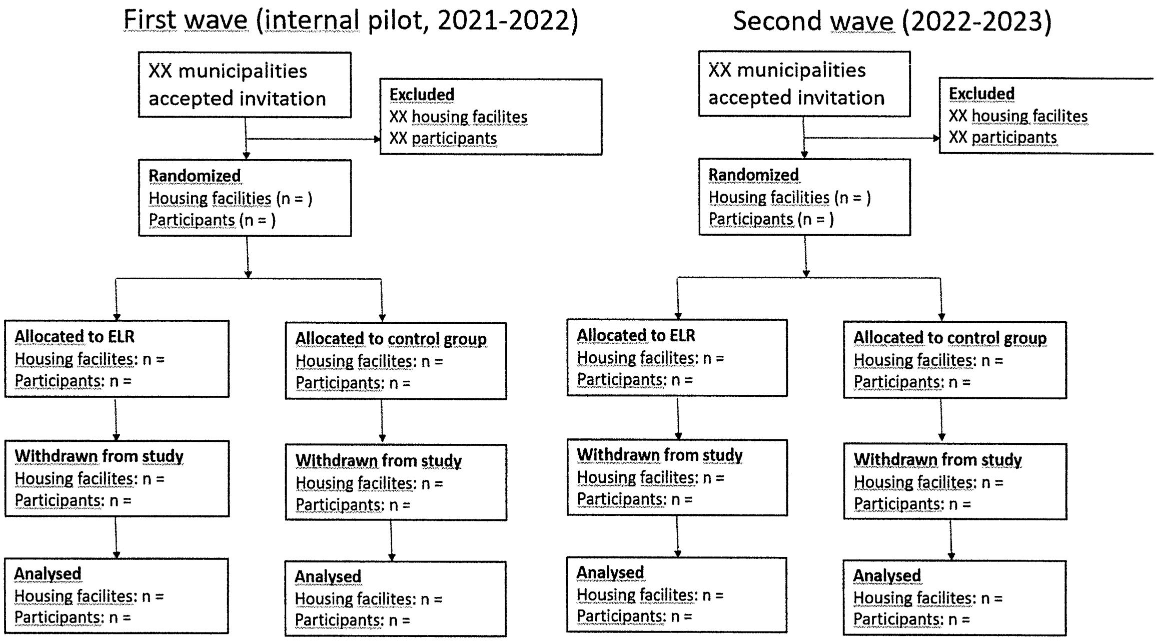

Figure 2. Draft of planned flow chart describing the enrollment, allocation and analysis of participants and housing facilities in the publication of the study.

Withdrawal/ Follow-up

Participants, housing staff, occupational therapists or municipalities may choose to withdraw from the study at any point. Reasons for withdrawal will be summarized for both groups, along with the

total frequency of participants lost to follow-up, in the publication of the study. Figure 2 shows draft of the information that will be reported in the flow chart.

Baseline patient characteristics

Baseline characteristics of both housing facility level and t individual participant level will be presented by group, in line with Consort Statement (ref). In table 1 below, baseline variables to be presented are listed.

Table 1. Variables to include in table of housing facility and patient characteristics. *Q1-Q3 – quartiles.*

**Variables at housing facility level**	**Comment/summary measure**
Type of housing facility	Number/percentage
**Variables at individual participant level**	
Sex	Number/percentage
Age	Median (Q1-Q3)
Civil status	Number/percentage
Highest education	Number/percentage
Ethnicity	Number/percentage borned in Sweden
Diagnosis	Number/percentage
Number of years living in housing facility	Median (Q1-Q3)

Analysis

Outcome definitions

Primary Outcome Measure: Recovering quality of life score at 6-months follow-up(2). An increase of points on the ReQoL score denotes improvement. In ReQoL-20, the minimum score is 0 and the maximum is 80, where 0 indicates poorest quality of life and 80 indicates highest quality of life.

Secondary Outcome Measure: Perceived personal recovery and daily functioning, assessed using the Recovery Assessment Scale - Domains & Stages (RAS-DS) (3) at 6 months follow-up. A total recovery score is gained from adding the scores for all items. The minimum score is 0 and the maximum score is 152.

Secondary Outcome Measure: Goal Attainment at 6-months follow-up

Goal-attainment will be measured within the intervention group only, using the Goal Attainment Scaling (GAS)(4). The scale has 5 points representing different levels of mastery of the individual participant's goal, from -2 to +2, where a score of minus (-2 and -1) represents less change than expected, 0 represents the expected level of change/attainment, and plus (+1 and +2) represents achievement of more change than expected. GAS uses T-scores, where scores are rescaled so that T-scores have a mean of 50 and a standard deviation of 10. If all of the goals meet the expected level of achievement, the GAS T-score will be 50. A high T-score (50 or above) is reflective of expected or higher goal-attainment.

Only within-group changes will be analyzed. The GAS measurement is measured at the 6-months follow-up.

Analysis methods

Primary Outcome Measure (ReQoL):

A mixed effects models will be used. The dependent variable will be the ReQoL 6 months follow-up measurement. A random intercept effect for housing facilities will be included in the model. Fixed effects independent variables will be *group* (ELR or control) and *baseline ReQoL measurement*. The baseline ReQoL will be adjusted for, on both an individual participant level and on an aggregated cluster level using the average of the baseline measurement within the corresponding housing facility(5). The treatment effect is presented as the baseline-adjusted group effect at 6 months follow-up, along with its 95 % confidence interval.

Secondary Outcome Measure RAS-DS:

The same mixed effects models as for ReQoL is planned to be used. The dependent variable will be the RAS-DS 6 months follow-up measurement. A random intercept effect for housing facilities will be included in the model. Fixed effects independent variables will be group (ELR or control) and baseline RAS-DS measurement. The baseline RAS-DS will be adjusted for, on both an individual participant level and on an aggregated cluster level using the average of the baseline measurement within the corresponding housing facility. The treatment effect is presented as the baseline-adjusted group effect at 6 months follow-up, along with its 95 % confidence interval

Secondary Outcome Measure GAS:

GAS will be evaluated using paired t-test, comparing the pre- and post-intervention t-score.

Model assumptions

Model assumption of normality for random effects and residual term and heterogeneity of variance within housing facility will be investigated graphically using the data from the interval pilot. Should assumption be deemed unlikely to hold, primarily the suitability of a logarithmic transformation of the outcome variable will be examined. Secondly, an ordinal cumulative link function model will be used to replace the mixed effects model if a fully parametrical model still is considered unsuitable. In such case, the study protocol and SAP will be updated with the planned changes in analysis method.

Sensitivity analyses

Analyses without imputation will be performed and reported in appendix of published study article.

Subgroup analyses

All analyses will be performed on men and women separately. Furthermore, sub-group analyses will be performed on:

autism - not autism

- alcohol-/drug addiction – not alcohol-/drug addiction, and

- psychosis-related – not psychosis related,

based on self-reported diagnosis/disability.

Missing data

For the primary intention-to-treat, missing data will be imputed using multiple imputation chained equations (MICE).

Additional analyses

All analyses will also be performed on the per-protocol population and its results reported in an appendix to study article. Any difference in conclusion between intention-to-treat analyses and per-protocol analyses will be discussed.

Statistical software

Statistical analyses will be made using R(6). The lmer function from the lme4 package (7) will be used to estimate the mixed effects models. P-values and confidence intervals will be calculated using Satterthwaite's degrees of freedom method, via the lmerTest package (8).

References

1. Gamble C, Krishan A, Stocken D, Lewis S, Juszczak E, Doré C, et al. Guidelines for the Content of Statistical Analysis Plans in Clinical Trials. JAMA. 2017 Dec 19;318(23):2337–43.

2. Keetharuth AD, Brazier J, Connell J, Bjorner JB, Carlton J, Buck ET, et al. Recovering Quality of Life (ReQoL): a new generic self-reported outcome measure for use with people experiencing mental health difficulties†. The British Journal of Psychiatry. 2018 Jan;212(1):42–9.

3. Hancock N, Scanlan JN, Honey A, Bundy AC, O'Shea K. Recovery Assessment Scale – Domains and Stages (RAS-DS): Its feasibility and outcome measurement capacity. Aust N Z J Psychiatry. 2015 Jul 1;49(7):624–33.

4. Kiresuk TJ, Smith A, Cardillo JE, editors. Goal Attainment Scaling: Applications, Theory, and Measurement. New York: Psychology Press; 2014. 336 p.

5. Hooper R, Forbes A, Hemming K, Takeda A, Beresford L. Analysis of cluster randomised trials with an assessment of outcome at baseline. BMJ. 2018 Mar 20;360:k1121.

6. R Core Team. R: A language and environment for statistical computing [Internet]. Vienna, Austria; 2021. Available from: https://www.R-project.org/

7. Pinheiro jose, Bates D, DebRoy S, Sarkar D. nlme: Linear and Nonlinear Mixed Effects Models [Internet]. R package version 3.1-152. 2021. Available from: https://CRAN.R-project.org/package=nlme

8. Kuznetsova A, Brockhoff PB, Christensen RHB. lmerTest Package: Tests in Linear Mixed Effects Models. Journal of Statistical Software. 2017 Dec 6;82:1–26.
